# Can breast MRI help in the management of women with breast cancer treated by neoadjuvant chemotherapy?

**DOI:** 10.1038/sj.bjc.6601710

**Published:** 2004-03-02

**Authors:** R M L Warren, L G Bobrow, H M Earl, P D Britton, D Gopalan, A D Purushotham, G C Wishart, J R Benson, W Hollingworth

**Affiliations:** 1 Department of Radiology, Cambridge Breast Unit, Addenbrooke's Hospital, Cambridge CB2 2QQ, UK; 2 Department of Histopathology, Cambridge Breast Unit, Addenbrooke's Hospital, Cambridge CB2 2QQ, UK; 3 Department of Medical Oncology, Cambridge Breast Unit, Addenbrooke's Hospital, Cambridge CB2 2QQ, UK; 4 Department of Surgery, Cambridge Breast Unit, Addenbrooke's Hospital, Cambridge CB2 2QQ, UK; 5 Department of Radiology, University of Washington, Seattle, WA 98103, USA

**Keywords:** breast cancer, breast MRI, disease response, diagnostic impact, neoadjuvant chemotherapy

## Abstract

Contrast-enhanced (CE) MRI was used to monitor breast cancer response to neoadjuvant chemotherapy. Patients underwent CE MRI before and after therapy, together with conventional assessment methods (CAM). CE MRI was carried out at 1.5 T in the coronal plain with 3D sequences before and after bolus injection. An expert panel determined chemotherapy response using both CE MRI and CAM. Histopathological response in the surgical specimen was then used to determine the sensitivity and specificity of CE MRI and CAM. In total, 67 patients with 69 breast cancers were studied (mean age of 46 years). Tumour characteristics showed a high-risk tumour population: median size 49 mm: histopathological grade 3 (55%): oestrogen receptor (ER) negative (48%). Histopathological response was as follows: – complete pathological response (pCR) 17%; partial response (pPR) 68%; no response (NR) 15%. Sensitivity of CAM for pCR or pPR was 98% (CI 91–100%) and specificity was 50% (CI 19–81%). CE MRI sensitivity was 100% (CI 94–100%), and specificity was 80% (CI 44–97%). The absolute agreement between assessment methods and histopathology was marginally higher for CE MRI than CAM (81 *vs* 68%; *P*=0.09). In 71%, CE MRI increased diagnostic knowledge, although in 20% it was judged confusing or incorrect. The 2nd MRI study significantly increased diagnostic confidence, and in 19% could have changed the treatment plan. CE MRI persistently underestimated minimal residual disease. In conclusion, CE MRI of breast cancer proved more reliable for predicting histopathological response to neoadjuvant chemotherapy than conventional assessment methods.

Breast cancer treatment has advanced over the past two decades, achieving a better outcome for women (Peto *et al*, 2000). One of several factors contributing to improved survival is chemotherapy, which is now widely used particularly for women under 50 years and those with high-grade ER negative or lymph node-positive tumours. When chemotherapy is given prior to definitive surgical treatment, there are various potential benefits (Valero *et al*, 2002):

- Primary tumour response provides evidence of responsiveness to the chosen chemotherapeutic agent and predicts local control (Chollet *et al*, 2002)
- A large tumour may become amenable to breast-conserving surgery (Makris *et al*, 1997; Fisher *et al*, 1998)
- An ineffective therapeutic agent may be changed to one with clinical effect, or surgery may be advanced, thereby saving cost and patient suffering (Sotiriou *et al*, 2002; Pusztai *et al*, 2003)

There is no evidence of benefit in overall or disease-free survival as a result of the use of chemotherapy in the neoadjuvant setting (Fisher *et al*, 1998; Green and Hortobagyi, 2002). Marginally statistically significant treatment-by-age interactions appear to be emerging for survival and disease-free survival, suggesting that younger patients may benefit from preoperative therapy, whereas the reverse may be true for older patients (Wolmark *et al*, 2001). It is logical to initiate systemic treatment early in those patients with a high risk of micro-metastatic disease and to monitor the response. Conventional tools to monitor response include measurement of maximum tumour diameter with callipers; loss of signs of inflammation and reduction in lymph node size. Serial core biopsy has been used to show changes in tissue morphology by standard histological criteria and more recently with molecular markers (Sotiriou *et al*, 2002; Pusztai *et al*, 2003). Response of the primary tumour or regional nodes may be monitored radiologically with mammography or ultrasound (Helvie *et al*, 1996; Vinnicombe *et al*, 1996; Huber *et al*, 2000); and newer imaging modalities (Mankoff *et al*, 1999; Tiling *et al*, 2001), such as contrast-enhanced (CE) MRI are being increasingly used. The literature contains many single institution reports of the use of breast MRI for monitoring tumour response or predicting tumour extent prior to surgery. Table 1

**Table 1:** Existing literature on the use of breast MRI to monitor response to chemotherapy

**Author**	**Origin**	**Year**	**Number**	**Measurement**	**Pathology response**
Knopp *et al* (1994)	Heidelberg, Germany	1994	Three case studies		Promising prediction
Gilles *et al* (1994)	Villejuif, France	1994	18		(15 out of 18) 83% Highly predictive of residual tumour
Abraham *et al* (1996)	Dallas & Little Rock, USA	1996	39		MRI correctly predicted residual disease in 30 out of 31 (97%)
Rieber *et al* (1997)	Ulm, Germany	1997	13		Underestimates residual tumour
Trecate *et al* (1998)	Milano, Italy	1998	27		Valid tool for monitoring response
Tsuboi *et al* (1999)	Kochi, Japan	1999	31		Valuable for predicting response and margins
Weatherall *et al* (2001)	Dallas, TX, USA	2001	22	Cc0.93MR, 0.63 mam	Accurate assessment of residual tumour
Nakamura *et al* (2001)	Tokyo, Japan	2001	15		Predict possibility of WLE. Mapping of dendritic tumours
Drew *et al* (2001)	Hull, UK	2001	17	Sensitivity 100%	Accurate method of predicting residual tumour
Esserman *et al* (2001)	UCSF	2001	33		Patterns of tumours identified
Partridge *et al* (2002)	UCSF	2002	52	R0.89, C0.60	MRI detected all residual disease
Balu-Maestro *et al* (2002)	Nice, France	2002	60 in 51 women		Valuable tool for size & multifocality prior to surgery
Rieber *et al* (2002)	Ulm, Germany	2002	58	Accuracy NR 83.3%, PR 82.4%	Good for assessing response to chemo, unreliable in CR cases where 66.7% had residual tumour
Cheung *et al* (2003)	Kwei San, Taiwan	2003	33		Useful to assess early response and predict tumour size change
Delille *et al* (2003)	Harvard, USA	2003	14	EFP_count_ accurate measure (*P*<.02)	Extraction flow product as marker of tumour response

summarises the literature to date on the use of breast MRI to monitor neoadjuvant chemotherapy. It can be seen from the right-hand column of this table that the findings have varied very substantially. Breast MRI appears to hold promise for this purpose, and provides measurements that are more accurate than clinical or ultrasound assessment (Davis *et al*, 1996; Esserman *et al*, 1999). It is important to know whether predictions of residual tumour are accurate. Breast MRI has good sensitivity for invasive tumour but relatively poor specificity in untreated disease (Mussurakis *et al*, 1996). The limitations are more apparent for invasive lobular carcinoma and *in situ* (Kinkel and Hylton, 2001). Contrast-uptake characteristics have been shown to be altered by chemotherapy, which may potentially diminish sensitivity and impair specificity (Rieber *et al*, 1997). Contrast uptake characteristics have been used to give an early indication of tumour response using innovative functional MR and computer methods (Padhani, 2002; Delille *et al*, 2003). It is therefore an open question whether CE MRI would be helpful in monitoring response, or whether limitations emerging from studies undertaken concurrent with this one would make it unreliable.

In this study, we have investigated the impact of breast MRI on decision-making. Almost all of our patients underwent mastectomy after neoadjuvant chemotherapy. It is therefore possible to correlate the extent of tumour detectable on breast MRI following chemotherapy with histopathology of the mastectomy specimen. The aims of our study were:

1. To determine whether CE MRI is an accurate indicator of tumour response to neoadjuvant chemotherapy in comparison to the histopathology gold standard.
2. To assess whether CE MRI increases the diagnostic confidence above that achieved by conventional assessment methods (CAM) (i.e. clinical examination, ultrasound and mammography)
3. To determine whether CE MRI can accurately identify a subset of patients who might be suitable for breast-conserving surgery following neoadjuvant chemotherapy.

## PATIENTS AND METHODS

### Patients and chemotherapy

The study had approval from the Local Research Ethics Committee for Cambridgeshire, and all women had given informed consent to participate. It is a retrospective review of 67 women with CE-MRI before and after neoadjuvant chemotherapy for breast cancer. The first 30 women were recruited between 1997 and 2001 to a prospective pilot study of CE MRI. The remaining 37 women were consecutively enroled once CE MRI monitoring became the standard of care at our centre in 2001. The decision to give neoadjuvant therapy was made by a multidisciplinary team, and in general included women less than 60 years with large, inflammatory or high-grade tumours. Chemotherapeutic regimens used in this series varied according to whether patients were treated in ongoing clinical trials (Appendix A). All cases had undergone mammography, ultrasound and core biopsy prior to the decision to treat with neoadjuvant chemotherapy, and the results of these tests were used by the team to determine which trials they should enter.

### Contrast enhanced MRI (CE MRI)

The patients had two MRI studies, at the start of treatment and prior to surgery. The initial MRI study was usually undertaken prior to the first course of chemotherapy except in occasional cases where limitations of access to MRI resulted in imaging after the first treatment had been given. The 2nd MRI study was performed after all chemotherapy and prior to surgery. The studies were undertaken on a 1.5 T magnet from GE Signa Echospeed, which was upgraded in 2002 with Excite software. The MRI technique is similar to that described for the MARIBS study (Brown *et al*, 2000), with the exceptions that the dose of gadolinium was 0.16 mmol kg^−1^ body weight, and no proton density study was undertaken in our study (Scanning protocol – Appendix B). MRI interpretation used a synthesis of dynamic and morphological features, including the pattern of contrast uptake and washout characteristics, and the studies were examined at the console to allow dynamic contrast enhancement curves at any point in the 3D volume of the dynamic acquisition. The studies were reported by radiologists and presented to the multidisciplinary team for decisions on patient management.

### Conventional methods of assessment

All patients had a clinical assessment prior to each of six cycles of chemotherapy. This included calliper measurement of tumour size, degree of inflammation and size of palpable axillary nodes. All patients underwent mammography, ultrasound and core biopsy for diagnosis. Ultrasound was carried out after three and six cycles of chemotherapy. Conventional breast imaging comprised mammography, ultrasound and core biopsy according to the guidelines of the United Kingdom National Health Service Breast Screening Programme (NHSBSP) (Wilson *et al*, 2001).

### Surgery

Surgery normally took place less than 4 weeks after the last course of chemotherapy. All patients underwent either mastectomy or wide local excision (WLE) with axillary lymph node dissection.

### Histopathological response

Response to treatment was scored by standard UICC (Therasse *et al*, 2000) categories of Complete Response (CR), Partial Response (PR), Non-Response (NR), Disease Progression (DP). The reports were produced at the time of clinical care without particular knowledge of the MRI findings. It should be noted that after chemotherapy the criteria for grading cannot be applied, and so when grades are given here they are based on core biopsy (Harris *et al*, 2003).

There are no recognised standard criteria for grading of histological response following primary chemotherapy. In this study, mastectomy specimens were handled according to the NHSBSP guidelines (NHSBSP publication No. 3, 1995). In addition, in those cases where no macroscopic tumour could be identified, extensive sampling of the original tumour site (as identified by imaging methods and clinical examination prior to treatment) was performed.

On histological examination, where distinct *in situ* and invasive tumour was identified this was morphologically compared with tumour seen on the original core biopsy. The ratio of tumour cell mass to surrounding tissue was evaluated. If degenerative changes within tumour cells were present this was noted. The nature of the tissue surrounding tumour cells was noted, viz was it a cellular stroma similar to that seen in the original biopsy or was it inflamed, hypocellular scar tissue.

Thus:

1. *No histological response*. Tumour resembling that in the original core biopsy was present and it extended within the breast tissue for the same or a greater distance than originally estimated by imaging and clinical examination. The nature of the tissue surrounding the tumour was cellular and did not resemble bland scar tissue.
2. *Complete histological response*. Absence of invasive carcinoma cells/or <1single, degenerate carcinoma cell/10 high-power fields. Absence of *in situ* carcinoma.
3. *Partial histological response*. Tumour resembling that in the original core biopsy but which was considerably reduced in size (it is necessary to approximate size parameters used in clinical response staging). Presence of residual microscopic foci of invasive carcinoma but with reduced tumour cell/stroma ratio or residual pure *in situ* carcinoma.

### Assessment of sensitivity, specificity of conventional assessment methods and CE MRI

Original records were used to score clinical response using CAM, CE MRI response and histopathological response, and from these sensitivity and specificity were calculated. Pathological response was regarded as the gold standard against which all evaluations of response were measured.

### Assessment of diagnostic impact and confidence of knowledge

**Figure 1:**
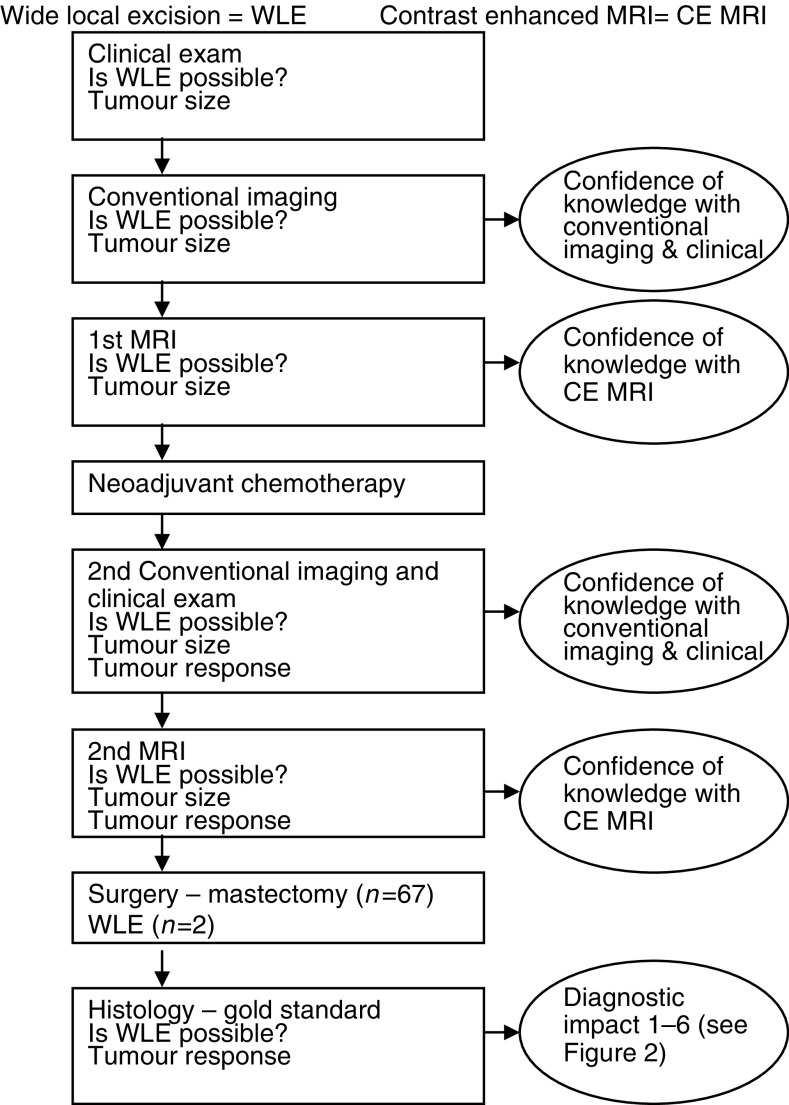
Algorithm for recording findings in the multidisciplinary meeting.

For the purposes of this study, all cases were reviewed by the multidisciplinary team and a research form completed to record the diagnostic impact. The review team consisted at minimum of a radiologist, a surgeon, an oncologist and a histopathologist, all expert in management of breast disease. The multidisciplinary review procedure is shown in Figure 1

**Figure 2:**
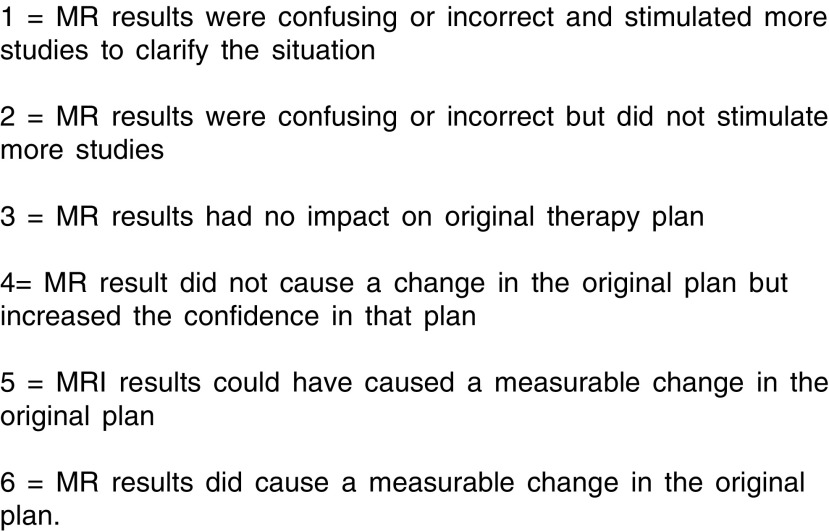
Value of including MRI imaging.

. The clinical notes were examined for the clinical findings recorded at the time of clinical care. The mammograms and ultrasound images with their original reports were presented as in a standard multidisciplinary team meeting. The MRI studies were presented to the clinical team using the images and reports taken at the time of clinical care of the patients. The multidisciplinary team were unaware of the histopathology findings when they interpreted the results of the MRI and other presurgical examinations. The pathology was presented from the histological report at the time of clinical care, and did not take particular account of MRI knowledge. The added value of MRI imaging was given a score 1…6 (Figure 2

) by the team, taking into account the additional contribution made by the MRI study beyond that available with conventional breast imaging (Shuman *et al*, 1985a,1985b). A visual analogue scale was used to record confidence of knowledge with conventional breast imaging alone and with the addition of MRI (Blanchard *et al*, 1997). Confidence of knowledge included knowledge of the size, the contour, the extent and the suitability for any form of surgery. The two measures, added value and confidence of knowledge are complementary, since confidence of knowledge is an important aspect of value to a clinician. These are subjective clinical judgements.

### Statistical analyses

Proportions in independent samples were compared using Fisher's exact test or linear by linear χ^2^ test as appropriate. Parametric and nonparametric paired comparisons of mean and median values were performed using the paired *t*-test and McNemar test, respectively. The relationship between initial tumour size and subsequent tumour response was assessed using one-way analysis of variance. Statistical significance was accepted at the conventional *P*<0.05 threshold.

## RESULTS

The study evaluated 69 cancers in 67 women including two bilateral cases. In all, 67 cancers were treated with mastectomy and two with WLE. The mean age of the patients at the time of the 1st MRI was 46.2 (range 28 to 62 years). On initial radiological examination, the median size of the 69 tumours was 49.0 mm (range 9–100 mm). The core biopsy demonstrated that six (9%) tumours were grade 1, 25 (36%) grade 2 and 38 (55%) grade 3. These therefore represent relatively large, poor prognosis cancers in younger women. In total, 36 (52%) tumours were oestrogen receptor (ER) positive, and 33 (48%) ER negative, 21 out of 26 (81%) patients where the progesterone receptor (PR) status was recorded were PR negative. At definitive surgery, patients had a median of 13 nodes retrieved (range 0–42); 17 patients (25%) had more than four nodes involved, 23 (33%) had between one and four nodes involved, and 29 (42%) were node negative. In all, 12 (17%) patients presented with inflammatory cancers, 31 (45%) were known to be multifocal or diffuse lesions by their imaging or clinical findings, and a further 16 (23%) lesions were close to the nipple–areolar complex. After the initial clinical and radiological evaluation, a total of 52 (75%) tumours were considered not suitable for breast conservation within the protocols of our unit, where skin involvement by inflammation, very large size, multifocality and proximity to the nipple–areolar complex are all viewed as reasons for not using breast-conserving surgery. The median period between the 1st and 2nd MR imaging was 119 days (range 48–165 days). The median period between the 2nd MR and surgery was 24 days (range 2–77 days). The neoadjuvant chemotherapy included cases in trials, notably Anglo-Celtic 2, and cases off trial with a variety of regimens (Appendix A).

### Estimation of response

Based on the gold standard histopathology findings, 12 lesions (17%) had complete response, 47 (68%) had partial response, 10 (15%) had no response following neoadjuvant chemotherapy (Table 2

**Table 2:** Comparison of clinical assessment with ultrasound and mammography and pathology rating of tumour response

		**Conventional assessment methods (CAM) Clinical examination+mammography and ultrasound**			
		**Complete response**	**Partial response**	**No response**	**Disease progression**
Pathology findings	Complete response	9	3	0	0
	Partial response	13	33	1	0
	No response	1	4	5	0
	Disease progression	0	0	0	0

). There was no significant association between the grade of the tumour and the extent of disease response following chemotherapy. In total, 24% (nine out of 38) of grade 3 tumours had complete response to chemotherapy compared to 12% (three out of 25) of grade 2 tumours and 0% (zero out of six) of grade 1 tumours (*P*=0.10). Four out of 36 (11%) ER-positive tumours exhibited complete response at pathology compared to six out of 21 (29%) of tumours that were ER- and PR negative (*P*=0.15). Lesions that did not respond or progressed after neoadjuvant chemotherapy were slightly, but nonsignificantly, larger than lesions that exhibited partial or complete response (mean size 56 *vs* 49 *vs* 48 mm; *P*=0.56). Tumour response was clearly related to the extent of node involvement. In all, 97% (28 out of 29) of lesions with no node involvement had complete or partial response compared to 87% (20 out of 23) of lesions with between one and four positive nodes and 65% (11/17) lesions with more than four involved nodes (*P*<0.01).

### Conventional assessment methods and MRI in the estimation of tumour response

The clinical assessment of tumour response agreed with the pathology assessment in 47 out of 69 tumours (68%) (Table 2). CAM underestimated the degree of response in four cases, and overestimated the degree of response in 18 cases. In one case, a tumour which was categorised as completely responsive based on the clinical data had no response evident at pathological examination. CAM correctly identified 58 out of 59 lesions with complete or partial response based on pathology findings (sensitivity 98%; 95% confidence interval (CI) 91–100%). However, five out of 10 tumours with no response were incorrectly categorised as complete or partial response on the clinical assessment (specificity 50%; CI 19–81%).

There was agreement between MRI findings and the pathology findings in 56 out of 69 lesions (81%) (Table 3

**Table 3:** Comparison of MRI and pathology rating of tumour response

		**CE MRI findings**			
		**Complete response**	**Partial response**	**No response**	**Disease progression**
Pathology findings	Complete response	12	0	0	0
	Partial response	10	37	0	0
	No response	1	1	7	1
	Disease progression	0	0	0	0

). The imaging findings underestimated the effect of chemotherapy in one case where disease appeared to have progressed on CE MRI, but where pathology results indicated no response. In a further 12 cases, MRI overestimated the response to therapy. The sensitivity (100%; CI 94–100%) and specificity (80%; CI 44–97%) of MRI for identifying complete or partially responsive tumours were higher than for the clinical assessment alone. The absolute agreement between MRI and pathology findings was marginally higher than the agreement between clinical assessment and pathology findings (81 *vs* 68%; *P*=0.09).

### Diagnostic confidence and therapeutic impact after MRI

In the majority of cases, the 2nd MRI study was judged to increase diagnostic knowledge (>70% see Table 4

**Table 4:** Potential diagnostic and therapeutic impact of 2nd MRI

**Impact of MRI**	***N* (%)**
*Diagnostic knowledge with 2nd MRI*	
Better	49 (71%)
Same	8 (12%)
Worse	12 (17%)
	
*Diagnostic value of 2nd MRI*	
Confusing or incorrect – extra studies required	0 (0%)
Confusing or incorrect – no extra studies	14 (20%)
No change in treatment plan	6 (9%)
No change in treatment plan – increased diagnostic confidence	36 (52%)
Could have caused change in treatment plan	10 (15%)
Caused change in the treatment plan	3 (4%)
	
*Diagnostic confidence of 2nd MRI*	
Without 2nd MRI (mean (s.d.))	5.8 (1.8)
With 2nd MRI (mean (s.d.))	7.9 (2.0)*
	
*Therapeutic impact of 2nd MRI*	
WLE possible based on surgery	22/69
MRI agrees with conventional assessment, WLE possible	10 (45%)
MRI agrees with conventional assessment, WLE NOT possible	5 (23%)
MRI disagrees with conventional assessment, suggests WLE possible	5 (23%)
MRI disagrees with conventional assessment, suggests WLE NOT possible	2 (9%)
	
WLE NOT possible based on surgery	47/69
MRI agrees with conventional assessment, WLE possible	4 (9%)
MRI agrees with conventional assessment, WLE NOT possible	35 (75%)
MRI disagrees with conventional assessment, suggests WLE possible	4 (9%)
MRI disagrees with conventional assessment, suggests WLE NOT possible	4 (9%)

* *P*<0.01, paired samples *t*-test for the difference in confidence pre- and post-2nd MRI.

). However, in a substantial proportion of cases (17% see Table 4) the 2nd MRI was judged to have reduced diagnostic knowledge. These 12 cases have been examined in greater detail – see Table 5A

**Table 5:** Cases in which the 2nd MRI study (A) reduced diagnostic knowledge *n*= 12 and (B) either did or had the potential to alter the treatment plan *n*=13

**Case**	**Age, years**	**Clinical size, mm at start of treatment**	**Grade**	**ER**	**Quadrants involved**	**Spaced out cells present**	**DCIS present**	**cR**	**mR**	**pR**	**Comments**
(A)											
1^a^,^b^	46	50	1	+	Multifoc	No	No	1	1	2	Underestimates residual tumour
2^a^,^b^	51	Unmeasurable	2	+	Unifocal	No	No	2	1	2	Underestimates residual tumour
6^a^,^b^	47	60+	3	+	Subareol	No	Yes	2	1	2	Underestimates residual tumour ILC
8^b^	41	Unmeasurable	3	−	Diffuse	No	Yes	1	2	2	Underestimates residual tumour
13^b^	54	40	2	+	Unifocal	No	Yes	1	1	2	Underestimates residual tumour
25^b^	39	60+	3	−	Diffuse	No	Yes	1	1	3	Underestimates residual tumour
29^a^,^b^	34	60+	3	−	Subareol	No	Yes	1	1	1	No contrast – failed injection CR report
30^a^	49	60+	2	+	Unifocal	Yes	Yes	2	1	2	Underestimates residual tumour DCIS
31^a^,^b^	51	60+	2	+	Multifoc	No	Yes	2	2	2	Underestimates residual tumour
32^b^	40	45	3	−	Multifoc	No	No	2	2	2	Underestimates residual tumour
34^a^	43	30	3	−	Unifocal	No	Yes	2	1	1	Residual DCIS
41^a^,^b^	34	60+	2	+	Multifoc	No	Yes	1	2	2	Underestimates residual tumour ILC
61^a^,^b^	47	60+	1	+	Multifoc	No	Yes	2	1	2	Underestimates residual tumour
63^a^,^b^	60	Unmeasurable	3	+	Diffuse	No	Yes	2	2	3	Underestimates residual tumour
66^a^,^b^	47	45	3	+	Subareol	No	Yes	2	2	2	Underestimates residual tumour
78^a^,^b^	28	60+	2	−	Unifocal	Yes	No	2	1	2	Underestimates residual tumour
											
(B)											
7	52	Unmeasurable	2	−	Unifocal	Yes	Yes	1	1	2	Could have endorsed WLE
14	49	40	3	−	Unifocal	No	Yes	2	2	2	Better estimate of residual tumour
21	47	60+	2	+	Subareol	No	Yes	3	2	2	Could have endorsed WLE
34	43	30	2	−	Unifocal	No	Yes	2	1	1	Could have endorsed WLE DCIS only
37	57	54	1	−	Multifoc	No	No	1	1	1	Could have endorsed WLE
44	56	40	3	−	Unifocal	No	No	1	2	2	Confirmed residual tumour
52	43	50	2	+	Subareol	No	Yes	2	3	3	Showed nonresponder. Skin thickening
55	43	30	3	+	Unifocal	No	Yes	1	1	1	Could have endorsed WLE
59	52	40	3	+	Unifocal	Yes	Yes	1	1	1	Could have endorsed WLE
62bilat	49	60+	2	+	Diffuse	No	Yes	2	2	2	Contralateral tumour shown
67	33	32	3	+	Multifoc	No	No	2	2	2	Did not confirm multifocality suspected on ultrasound. Could have endorsed WLE
71	37	55	2	−	Subareol	No	No	1	1	1	Could have endorsed WLE

Grade Bloom & Richardson grades 1, 2 & 3 (Elston and Ellis, 1991) oestrogen receptor=ER positive (+), negative (−).

Ductal carcinoma *in situ*=DCIS, invasive lobular carcinoma=ILC. Quadrants – unifocal, multifocal, diffuse, subareolar position.

cR=clinical response, mR=MRI response, pR=pathological response 1=complete response, 2=partial response, 3=static disease, 4=disease progression. Wide local excision WLE.

a Cases in which the 2nd MRI study reduced diagnostic knowledge *n*=12.

b Cases in which the MRI findings were thought to be confusing or incorrect *n*=14.

.

In the majority of patients the 2nd MRI study did not change the treatment plan but did increase diagnostic confidence (52%, Table 4). This confidence was clearly valued by clinicians. Again, in a substantial minority of cases (20%, Table 4) the MRI findings were thought to be confusing or incorrect. In the majority of these cases, the problem stemmed from the MRI study underestimating the amount of residual tumour by suggesting a complete response when, in fact, there were residual invasive tumour cells or DCIS. These cases have been examined more closely and the findings set out in Table 5A. In a further 19% of cases (Table 4) the 2nd MRI either did, or had the potential to alter the treatment plan. These cases have been summarised in Table 5b. The reasons included a contralateral nonpalpable tumour, complete response that could have allowed breast conservation, demonstration of nonresponse, demonstration of a single focus where multifocality was suspected. The mean diagnostic confidence for all patients significantly increased from 5.8 pre-2nd MRI to 7.9 post-2nd MRI (*P*<0.01; Table 4).

Surgical findings indicated that WLE would not have been possible following neoadjuvant chemotherapy in 47 out of 69 lesions (Table 4). In 35 out of these 47 cases, both conventional assessment and MRI correctly predicted the need for mastectomy. The remaining 12 cases were evenly split between cases where conventional assessment incorrectly predicted WLE was possible (4), cases where MRI incorrectly predicted WLE was possible (4), and cases were both presurgery evaluations incorrectly suggested that breast-conserving surgery was possible (4).

In all, 10 out of the 22 lesions (46%) where surgical findings demonstrated that WLE would have been feasible were correctly identified by both conventional assessment and MRI (Table 4). In five cases, MRI correctly contradicted the conventional assessment by suggesting that WLE was possible. However, in the remaining seven cases MRI suggested that WLE was possible when surgical findings indicated that it was not. The overall agreement with surgical findings regarding the feasibility of WLE was 74% (51 out of 69) for conventional assessment and 78% (54out of 69) for MRI (*P*=0.44). Cases where the 2nd MRI study disagreed with surgical findings regarding the possibility of WLE are summarised in more detail in Table 6

**Table 6:** Cases where the 2nd MRI study disagreed with surgical findings regarding the possibility of WLE

**Case no.**	**Age in years**	**Radiological size, mm**	**Core biopsy grade**	**ER**	**Initial assessment allows WLE**	**MRI2 indicates WLE possible**	**Final pathology indicates WLE possible**	**DCIS present in final pathology**	**Spaced out cells present in final pathology**	**Comments**
1	46	60	1	+	No	No	Yes	No	No	15 mm unifocal
4	38	70	2	+	No	No	Yes	Yes	No	22 mm unifocal
14	49	34	3	−	Yes	No	Yes	Yes	No	31 mm unifocal
22	44	19	3	−	No	No	Yes	No	No	No residual tumour
27	45	35	3	+	No	No	Yes	No	No	No residual tumour
31	51	58	2	+	No	No	Yes	Yes	No	25 mm unifocal
62	49	39	2	+	No	No	Yes	Yes	No	20 mm unifocal
2	51	55	2	+	Yes	Yes	No	No	No	Multifocal
6	47	53	3	+	No	Yes	No	Yes	No	Widespread residual
10	41	51	2	+	No	Yes	No	Yes	No	41 mm residue
11	60	38	3	−	No	Yes	No	No	No	Mass+scattered foci
13	54	34	2	+	Yes	Yes	No	Yes	No	Multifocal
29	34	100	3	−	No	Yes	No	Yes	No	CR but inflammatory at outset
38	43	60	2	+	No	Yes	No	Yes	No	Widespread foci
61	47	50	1	+	No	Yes	No	Yes	No	Multifocal

.

## DISCUSSION

**Figure 3:**
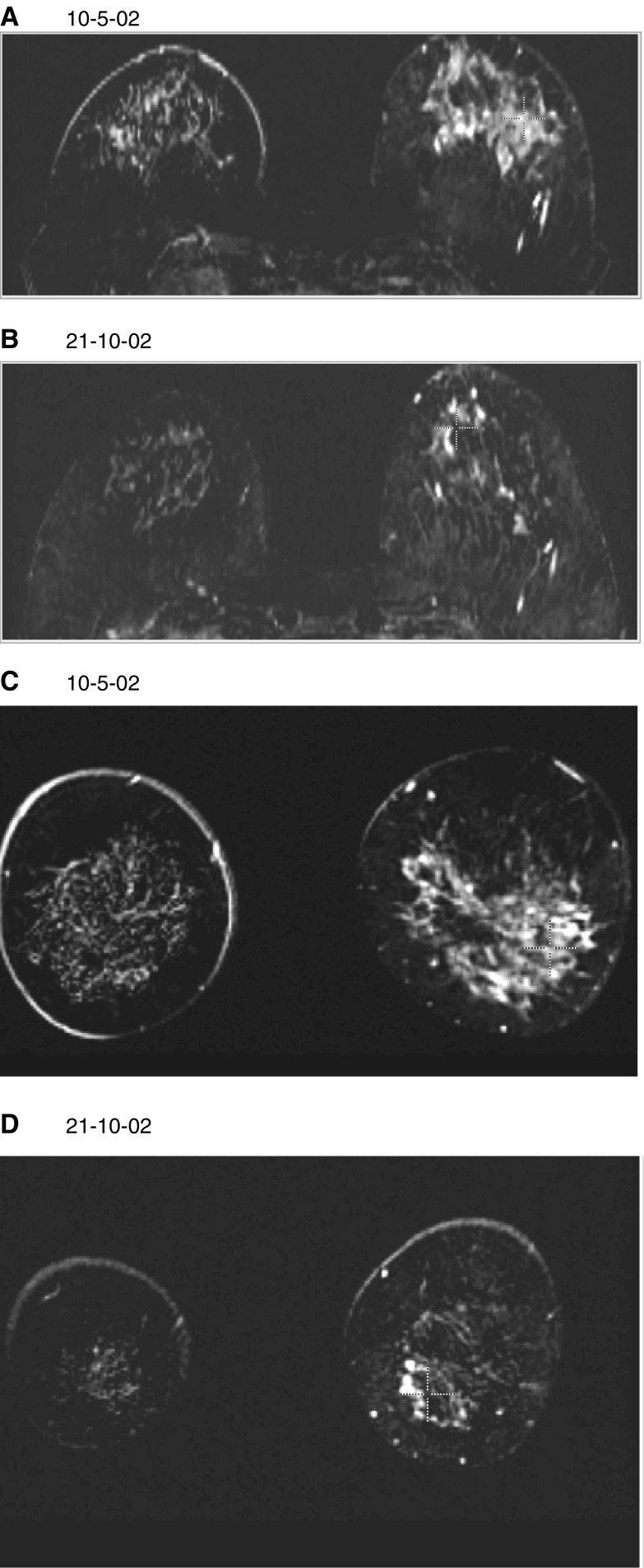
A subtracted image of a large tumour of the left breast before and after neoadjuvant chemotherapy. (**A**) and (**B**) reconstructed in the axial plane, (**C**) and (**D**) in the coronal plane. The patient was aged 52 years, had a clinically immeasurable tumour involving the nipple and so not suitable for WLE. It was grade 3, ER positive. She was treated with adriamycin and cyclophosphamide 6 cycles, off-study. She had a complete clinical response, but final histology showed residual grade 3 tumour with associated high-grade DCIS. The MRI studies show the considerable response, but correctly predicted residual tumour over a substantial volume, confirmed as over 60 mm on final histology.

To achieve the objectives of neoadjuvant timing in the use of chemotherapy, it is necessary to understand the available preoperative methods of assessing response. Assessment of response to permit conservation surgery and to judge the effectiveness of the chemotherapy and the biological responsiveness of tumours are both important. Clinical methods have been unreliable (El-Didi *et al*, 2000), and there are clear limitations in the power of mammography (Huber *et al*, 2000) or ultrasound (for this purpose, ultrasound is widely used, but very poorly researched) to provide accurate information. CE breast MRI has attractions for this purpose, since it has high sensitivity for invasive tumour and is dependent on increase in vessel density and abnormal diffusion properties of tumour blood vessels for its mode of visualisation. It is essentially a tomographic technique, able to give attractive 3D images that can aid surgeons in planning their operative procedures (Figure 3

).

There have been a number of studies testing CE breast MRI for this purpose, which have been summarised in Table 1. The conclusions of these studies have varied considerably and several of them have an up-beat approach that may give surgeons and oncologists false expectations of the capacity of the technique to be reliable in all the aspects needed for planning treatment. Our study is from a single multidisciplinary team in two hospitals, with clinical work of several experts in each discipline. We have used the contemporary reports and notes of the cases to assess the impact, real or potential, on clinical care provided by a CE MRI study at the start and end of neoadjuvant chemotherapy. We have used the histopathological response as the gold standard and since most of our patients had mastectomy, we have the potential to judge whether WLE could have been achieved. By using the full multidisciplinary team in evaluating each case, we hope to have avoided the bias that might ensue from radiologists alone assessing their value. Diagnostic and therapeutic impact methodology has been used for assessment of other new diagnostic procedures (Shuman *et al*, 1985b) including MRI (Hollingworth *et al*, 2000) for other purposes. We have moulded this methodology to apply to the present clinical scenario, for example by including an extra category – ‘MRI results could have caused a measurable change in the original plan’.

A few patients had their MRI study after the first cycle of chemotherapy was given, but these cases have not been excluded because the end points in this analysis relate to the presurgical MRI findings. The range of length between the 2nd MRI and surgery is determined by difficulties with scheduling of both MRI studies and reconstructive surgery in our hospital. The range of times between 1st and 2nd MRI studies is determined by the different regimens and patient episodes such as depression of white count which may have delayed cycles of chemotherapy. Whether these variations are important to the end points of this analysis is a matter of speculation, but they relate to pressures that would apply to real-life use of MRI out of the study setting. Our patients received a variety of chemotherapeutic regimens but this should make our CE MRI evaluation generalisable to actual clinical practice where chemotherapy choices vary. The analysis of the MRI study is not affected by the different chemotherapy regimens.

We have made the main comparison of sensitivity and specificity between clinical examination coupled with conventional breast imaging (mammography and breast ultrasound) and CE MRI. CE MRI had both better sensitivity and specificity than clinical measures in conjunction with conventional breast imaging for detecting complete or partial response. The sensitivity of conventional methods is high, but there are serious limitations in specificity. In all, 50% (five out of 10) of patients with no response on pathology were incorrectly classified as partial or complete responders on clinical assessment, mammography and ultrasound. The specificity of CE MRI was higher (eight out of 10), but, due to the small number of patients who did not respond to chemotherapy, this result did not reach statistical significance. Furthermore, CE MRI still gives erroneous results in a number of cases. This is demonstrated in Table 5a where it is seen that the commonest limitation of the value of CE MRI is to underestimate residual tumour when compared with final pathology. Several authors (Gilles *et al*, 1994; Drew *et al*, 2001; Weatherall *et al*, 2001; Partridge *et al*, 2002) have found very good prediction of residual tumour by breast MRI, whereas Rieber in two studies had findings similar to ours (Rieber *et al*, 1997, 2002). We wish to seek the explanation for this in greater detail, and will do so by a separate publication studying the dynamic analysis compared with detailed pathology. Many of the cases with small amounts of residual tumour have islands of invasive cells or of DCIS spread across the area previously occupied by the treated tumour. The contrast uptake of the CE MRI did not distinguish this minimal residual disease (MRD) from any background uptake within the glandular tissue of the breast. The chemotherapeutic agents attenuate the contrast uptake characteristics, rendering the curves on some occasions benign in appearance. Clinical assessment of response is based on the size of the palpable mass and the clinical evidence of reduction in lymphoedema or in lymph node size. CE MRI was shown here to be better than these clinical findings even when they are backed up by ultrasound.

The cases in which CE MRI gave added value are shown in Table 5b. These are demonstration of unsuspected contralateral tumour, endorsement of a decision for WLE, better demonstration of nonresponse by showing skin thickening, better estimate of residual tumour and proof of unifocal disease. In Table 6, we have scrutinised the performance of CE MRI in predicting the possibility of WLE after neoadjuvant chemotherapy. It was a surprise to us that so few cases were suitable for breast-conserving surgery from the start, and we do not have the power here to show whether MRI was helpful in a significant way when only the cases which from the outset could have been suitable are considered.

It has been shown that pathological response is predictive of survival (Kuerer *et al*, 1999; Amat *et al*, 2002; Chollet *et al*, 2002) and that outcome is predicted also by tumour grade and nodal involvement. It would be useful to know whether complete response on MRI is predictive of outcome, even when it fails to identify isolated foci of malignant cells or residual DCIS. If response on MRI were to be predictive of survival or disease-free survival, then this would be a test that gave predictive information prior to surgery and full pathology. Many more cases than the present study would be required to answer this question.

Within this series, the grade 3 and ER negative tumours tended to have a slightly better probability of complete response than the lower grade, ER-positive tumours, however neither of these differences reached statistical significance. A factor to consider here is the under staging that occurs with core biopsy, where 70% of cases have been shown to be upstaged between core biopsy and definitive surgical histology (Harris *et al*, 2003). After chemotherapy, we do not have satisfactory grading, and so the grades discussed here are all from core biopsy. The response rate that has been recorded cannot be appraised since it is dependent on the case selection for use of neoadjuvant chemotherapy and the different regimens in use, neither of which have been made standard in this study. We are looking at the effectiveness of CE MRI as a tool for monitoring, not at response rates in themselves. Our study shows that our MRI findings had very serious limitations in predicting the possibility of undertaking wide local excision after neoadjuvant chemotherapy, and that the shortfall was in both directions and not predictable from any particular characteristics of the cases. This study also shows how inaccurate clinical assessment of tumour response is, even when enhanced by ultrasound or mammography.

Even though our study has shown definite limitations to the reliability of MRI, the clinical team found that it provided valuable additional information. The problems with CE MRI are sufficient to continue to seek improved methods of assessing tumour response. Functional MRI, PET scanning and molecular imaging are emerging techniques that may offer more accurate information.

## CONCLUSION

Our study suggests that CE MRI may have better sensitivity and specificity for predicting complete or partial response than clinical examination with conventional imaging. Moreover, the absolute agreement between MRI and pathology findings was marginally higher than the agreement between clinical assessment and pathology findings. CE breast MRI is robust in predicting nonresponse and disease progression. Our clinicians valued the visual images that are obtained which show tumour response. They feel that they give additional information – probably by giving a better measure of size and providing images that help them to understand the permeative properties of the tumour. In our series, MRI persistently underestimates residual malignancy where the tumour is not a single mass, and this may be a problem in some cases when it is used to predict the possibility of WLE.

## Appendix A

See Tables A1

**Table A1:** Chemotherapy regimens in use

	***n***
Doxorubicin/cyclophosphamide six cycles	41
Doxorubicin/docetaxel six cycles	21
Epirubicin four cycles then CMF four cycles	2
Epirubicin four cycles	2
Docetaxel/Herceptin	1
Total	67

Clinical trials (1997–2003): *Anglo-Celtic 2 Study*: doxorubicin/cyclophosphamide *vs* doxorubicin/docetaxel six cycles (Evans *et al*, 2002). _ Docetaxel/Herceptin expanded access phase 4 assessment (Howell *et al*, 2002). Standard off study treatment: doxorubicin/cyclophosphamide six cycles.

and A2

**Table A2:** Cases included in the study

**Case**	**Age**	**Size**	**Infl**	**Gr**	**ER**	**PgR**	**Hist**	**Trial**	**cR**	**mR**	**pR**
01	46	60	n	1	+		IDC	AC2	cCR	mPR	pPR
02	51	55	n	2	+		IDC	AC2	cPR	mPR	pPR
03	51	65	n	3	+		IDC		cCR	mPR	pPR
50	40	55	n	3	−	−	IDC		cCR	mPR	pPR
46	44	95	n	3	−	−	IDC		cPR	mPR	pPR
04	38	70	n	2	+		IDC	AC2	cPR	mPR	pPR
05	55	45	n	1	+		IDC		cPR	mPR	pPR
06	47	53	n	3	+		ILC	AC2	cPR	mCR	pPR
07	52	70	n	2	−	0	IDC	AC2	cCR	mCR	pPR
51	31	48	n	2+	−	−	IDC		cPR	mCR	pCR
08	41	70	y	3	−	−	IDC	AC2	cCR	mPR	pPR
10	41	51	n	2	+		ILC		cPR	mPR	pPR
11	60	38	n	3	−	+	IDC		cPR	mPR	pPR
12	56	88	y	1	+		IDC		cNR	mNR	pNR
42	61	65	n	3	−	−	IDC		cNR	mNR	pNR
13	54	34	n	2	+		IDC	AC2	cCR	mCR	pPR
41	34	69	y	2	+		ILC	AC2	cCR	mPR	pPR
14	49	34	n	3	−	−	IDC		cPR	mPR	pPR
40	61	34	n	3	+		IDC	AC2	cPR	mCR	pPR
15	54	32	n	3	+		IDC	AC2	cCR	mPR	pPR
16	39	53	y	3	−	0	IDC		cNR	mDP	pNR
59	52	55	n	3	+		IDC	AC2	cCR	mCR	pCR
17	49	70	y	2	−	0	IDC		cPR	mPR	pPR
18	47	49	n	3	−	−	IDC	AC2	cPR	mPR	pPR
54	49	69	y	3	−	−	IDC	AC2	cCR	mCR	pCR
55	43	35	n	3	+		IDC	AC2	cCR	mCR	pCR
19	35	50	n	3	−	+	IDC	AC2	cPR	mPR	pPR
20	45	90	y	2	+		IDC		cPR	mPR	pPR
21	47	45	n	2	+		IDC	AC2	cNR	mPR	pPR
47	30		n	3	−	+	IDC		cPR	mNR	pNR
22	45	19	y	3	−	−	IDC	AC2	cPR	mCR	pCR
23	49	40	n	3	−	−	IDC	AC2	cPR	mPR	pPR
43	50	46	n	3	−	−	IDC	AC2	cPR	mPR	pPR
39	44	39	n	3	+		IDC	AC2	cCR	mCR	pCR
25	39	45	n	3	−	−	IDC	AC2	cCR	mCR	pNR
26	33	26	n	3	−	−	IDC	AC2	cPR	mPR	pCR
27	45	35	n	3	+		IDC		cCR	mCR	pCR
57	47	36	n	3	−	−	IDC	AC2	cPR	mNR	pNR
29	34	100	y	3	−	−	IDC/ILC		cCR	mCR	pCR
30	49	54	n	2	+		IDC	AC2	cPR	mCR	pPR
31	51	58	n	2	+		IDC	AC2	cPR	mPR	pPR
32	40	44	n	3	−	−	IDC	AC2	cPR	mPR	pPR
33	41	90	y	3	+		IDC		cCR	mPR	pPR
34	43	30	n	3	−	0	IDC	AC2	cPR	mCR	pCR
58	59	66	n	3	−	−	IDC		cPR	mPR	pPR
35	49	66	n	2	+		IDC		cNR	mNR	pNR
36	45	49	n	2	+		ILC	AC2	cPR	mPR	pPR
61	45	50	n	3	+		IDC	AC2	cPR	mCR	pPR
37	57	64	n	2	−	−	ILC	AC2	cCR	mCR	pCR
38	43	60	y	2	+		IDC	AC2	cCR	mPR	pPR

**Key to column headings**: Size=radiological (mammography and ultrasound) size at diagnosis, inflamm=inflammatory changes present, grade= Bloom and Richardson nuclear grade (Elston and Ellis, 1991) on core biopsy, ER=oestrogen receptor status on core biopsy, PgR=progesterone receptor status, Histology=core biopsy (CB) histology, IDC Invasive ductal carcinoma, ILC=invasive lobular carcinoma, Chemo=neoadjuvant chemotherapy regimen, cR=clinical response, mR=MRI response, pR=pathological response, CR=complete response, PR=partial response, NR=nonresponder, DP=disease progression. AC2=Anglo-Celtic 2 trial.

.

## Appendix B

See Table B1

**Table B1:** MR pulse sequence protocol for a breast MR examination in use for this study

**Sequence type**	**Repetition time TR (ms)**	**Echo time TE (MS) 1.5 T**	**Flip angle (^0^)**	**Matrix size**	**Number of slices**	**Field of view (FOV) (MM)**	**Pixel size (mm x mm)**	**Number of averages**	**Acquisition time (min:s)**
Precontrast, high-resolution 3D T_1_-weighted	8.9	4.2	35	512 × 384	60	340	0.66 × 0.89	1	2
Dynamic contrast enhanced, 3D T_1_-weighted^a^ 2 sequences before contrast injection and 6 after	8.8	4.2	35	256 × 256^b^	480	340	1.33 × 1.33^b^	1	8 × 1.10
Postcontrast, high-resolution 3D T_1_-weighted with fat suppression	18.9	4.2	35	512 × 384	60	340	0.66 × 0.89	1	5

a Contrast injection was 0.16 mmol kg^−1^ body weight with an injection speed of 3 ml s^−1^, given after the second precontrast dynamic sequence.

b The matrix size is optimised to ensure that the acquisition time of each 3D image is 70 s.

.

## References

[bib1] Abraham DC, Jones RC, Jones SE, Cheek JH, Peters GN, Knox SM, Grant MD, Hampe DW, Savino DA, Harms SE (1996) Evaluation of neoadjuvant chemotherapeutic response of locally advanced breast cancer by magnetic resonance imaging. Cancer 78: 91–100 8646731 10.1002/(SICI)1097-0142(19960701)78:1<91::AID-CNCR14>3.0.CO;2-2

[bib2] Amat S, Penault-Llorca F, Cure H, Le Bouedec G, Achard JL, Van Praagh I, Feillel V, Mouret-Reynier MA, Dauplat J, Chollet P (2002) Scarff–Bloom–Richardson (SBR) grading: a pleiotropic marker of chemosensitivity in invasive ductal breast carcinomas treated by neoadjuvant chemotherapy. Int J Oncol 20: 791–796 11894126

[bib3] Balu-Maestro C, Chapellier C, Bleuse A, Chanalet I, Chauvel C, Largillier R (2002) Imaging in evaluation of response to neoadjuvant breast cancer treatment benefits of MRI. Breast Cancer Res Treat 72: 145–152 12038705 10.1023/a:1014856713942

[bib4] Blanchard TK, Bearcroft PW, Dixon AK, Lomas DJ, Teale A, Constant CR, Hazleman BL (1997) Magnetic resonance imaging or arthrography of the shoulder: which do patients prefer? Br J Radiol 70: 786–790 9486041 10.1259/bjr.70.836.9486041

[bib5] Brown J, Buckley D, Coulthard A, Dixon AK, Dixon JM, Easton DF, Eeles RA, Evans DG, Gilbert FG, Graves M, Hayes C, Jenkins JP, Jones AP, Keevil SF, Leach MO, Liney GP, Moss SM, Padhani AR, Parker GJ, Pointon LJ, Ponder BA, Redpath TW, Sloane JP, Turnbull LW, Walker LG, Warren RM (2000) Magnetic resonance imaging screening in women at genetic risk of breast cancer: imaging and analysis protocol for the UK multicentre study [In Process Citation]. Magn Reson Imaging 18: 765–776 11027869 10.1016/s0730-725x(00)00167-3

[bib6] Cheung YC, Chen SC, Su MY, See LC, Hsueh S, Chang HK, Lin YC, Tsai CS (2003) Monitoring the size and response of locally advanced breast cancers to neoadjuvant chemotherapy (weekly paclitaxel and epirubicin) with serial enhanced MRI. Breast Cancer Res Treat 78: 51–58 12611457 10.1023/a:1022153327339

[bib7] Chollet P, Amat S, Cure H, de Latour M, Le Bouedec G, Mouret-Reynier MA, Ferriere JP, Achard JL, Dauplat J, Penault-Llorca F (2002) Prognostic significance of a complete pathological response after induction chemotherapy in operable breast cancer. Br J Cancer 86: 1041–1046 11953845 10.1038/sj.bjc.6600210 PMC2364175

[bib8] Davis PL, Staiger MJ, Harris KB, Ganott MA, Klementaviciene J, McCarty Jr KS, Tobon H (1996) Breast cancer measurements with magnetic resonance imaging, ultrasonography, and mammography. Breast Cancer Res Treat 37: 1–9 10.1007/BF01806626 8750522

[bib9] Delille J, Stanetz P, Yeh E, Halpern E, Kopans D, Garrido L (2003) Invasive ductal breast carcinoma response to neoadjuvant chemotherapy: noninvasive monitoring with functional MR imaging – pilot study. Radiology 228: 63–69 12775851 10.1148/radiol.2281011303

[bib10] Drew PJ, Kerin MJ, Mahapatra T, Malone C, Monson JR, Turnbull LW, Fox JN (2001) Evaluation of response to neoadjuvant chemoradiotherapy for locally advanced breast cancer with dynamic contrast-enhanced MRI of the breast. Eur J Surg Oncol 27: 617–620 11669587 10.1053/ejso.2001.1194

[bib11] El-Didi MH, Moneer MM, Khaled HM, Makarem S (2000) Pathological assessment of the response of locally advanced breast cancer to neoadjuvant chemotherapy and its implications for surgical management. Surg Today 30: 249–254 10752778 10.1007/s005950050054

[bib12] Elston CW, Ellis IO (1991) Pathological prognostic factors in breast cancer. I. The value of histological grade in breast cancer: experience from a large study with long-term follow-up. Histopathology 19: 403–410 1757079 10.1111/j.1365-2559.1991.tb00229.x

[bib13] Esserman L, Hylton N, Yassa L, Barclay J, Frankel S, Sickles E (1999) Utility of magnetic resonance imaging in the management of breast cancer: evidence for improved preoperative staging [In Process Citation]. J Clin Oncol 17: 110–119 10458224 10.1200/JCO.1999.17.1.110

[bib14] Esserman L, Kaplan E, Partridge S, Tripathy D, Rugo H, Park J, Hwang S, Kuerer H, Sudilovsky D, Lu Y, Hylton N (2001) MRI phenotype is associated with response to doxorubicin and cyclophosphamide neoadjuvant chemotherapy in stage III breast cancer. Ann Surg Oncol 8: 549–559 11456056 10.1007/s10434-001-0549-8

[bib15] Evans T, Gould A, Foster L, Crown J, Leonard R, Mansi J, Anglo-Celtic Collaborators (2002) Phase III randomised trial of adriamycin (A) and docetaxel (D) versus A and cyclophosphamide (C) as primary medical therapy (PMT) in women with breast cancer: an ACCOG study. J Clin Oncol 21, Abs 136

[bib16] Fisher B, Bryant J, Wolmark N, Mamounas E, Brown A, Fisher ER, Wickerham DL, Begovic M, DeCillis A, Robidoux A, Margolese RG, Cruz Jr AB, Hoehn JL, Lees AW, Dimitrov NV, Bear HD (1998) Effect of preoperative chemotherapy on the outcome of women with operable breast cancer. J Clin Oncol 16: 2672–2685 9704717 10.1200/JCO.1998.16.8.2672

[bib17] Gilles R, Guinebretiere JM, Toussaint C, Spielman M, Rietjens M, Petit JY, Contesso G, Masselot J, Vanel D (1994) Locally advanced breast cancer: contrast-enhanced subtraction MR imaging of response to preoperative chemotherapy. Radiology 191: 633–638 8184039 10.1148/radiology.191.3.8184039

[bib18] Green M, Hortobagyi GN (2002) Neoadjuvant chemotherapy for operable breast cancer. Oncology (Huntingt) 16: 871–884 889; discussion 889–90, 892–4, 897–8 12164555

[bib19] Harris GC, Denley HE, Pinder SE, Lee AH, Ellis IO, Elston CW, Evans A (2003) Correlation of histologic prognostic factors in core biopsies and therapeutic excisions of invasive breast carcinoma. Am J Surg Pathol 27: 11–15 12502923 10.1097/00000478-200301000-00002

[bib20] Helvie MA, Joynt LK, Cody RL, Pierce LJ, Adler DD, Merajver SD (1996) Locally advanced breast carcinoma: accuracy of mammography versus clinical examination in the prediction of residual disease after chemotherapy. Radiology 198: 327–332 8596826 10.1148/radiology.198.2.8596826

[bib21] Hollingworth W, Todd CJ, Bell MI, Arafat Q, Girling S, Karia KR, Dixon AK (2000) The diagnostic and therapeutic impact of MRI: an observational multi-centre study. Clin Radiol 55: 825–831 11069736 10.1053/crad.2000.0546

[bib22] Howell A, Houston S, Coleman R, Earl H, Wroath A, Miles D (2002) Safety and efficacy of Herceptin (H) alone or in combination with a taxoid (T) in HER2-positive metastatic breast cancer (MBC): the UK Expanded Access Programme (EAP). J Clin Oncol 21, Abs 1855

[bib23] Huber S, Wagner M, Zuna I, Medl M, Czembirek H, Delorme S (2000) Locally advanced breast carcinoma: evaluation of mammography in the prediction of residual disease after induction chemotherapy. Anticancer Res 20: 553–558 10769724

[bib24] Kinkel K, Hylton NM (2001) Challenges to interpretation of breast MRI. J Magn Reson Imaging 13: 821–829 11382939 10.1002/jmri.1117

[bib25] Knopp MV, Brix G, Junkermann HJ, Sinn HP (1994) MR mammography with pharmacokinetic mapping for monitoring of breast cancer treatment during neoadjuvant therapy. Magn Reson Imaging Clin N Am 2: 633–658 7489314

[bib26] Kuerer HM, Newman LA, Smith TL, Ames FC, Hunt KK, Dhingra K, Theriault RL, Singh G, Binkley SM, Sneige N, Buchholz TA, Ross MI, McNeese MD, Buzdar AU, Hortobagyi GN, Singletary SE (1999) Clinical course of breast cancer patients with complete pathologic primary tumor and axillary lymph node response to doxorubicin-based neoadjuvant chemotherapy. J Clin Oncol 17: 460–469 10080586 10.1200/JCO.1999.17.2.460

[bib27] Makris A, Powles TJ, Dowsett M, Osborne CK, Trott PA, Fernando IN, Ashley SE, Ormerod MG, Titley JC, Gregory RK, Allred DC (1997) Prediction of response to neoadjuvant chemoendocrine therapy in primary breast carcinomas. Clin Cancer Res 3: 593–600 9815725

[bib28] Mankoff DA, Dunnwald LK, Gralow JR, Ellis GK, Drucker MJ, Livingston RB (1999) Monitoring the response of patients with locally advanced breast carcinoma to neoadjuvant chemotherapy using [technetium 99 m]-sestamibi scintimammography. Cancer 85: 2410–2423 10357412

[bib29] Mussurakis S, Buckley DL, Coady AM, Turnbull LW, Horsman A (1996) Observer variability in the interpretation of contrast enhanced MRI of the breast. Br J Radiol 69: 1009–1016 8958017 10.1259/0007-1285-69-827-1009

[bib30] Nakamura S, Kenjo H, Nishio T, Kazama T, Do O, Suzuki K (2001) 3D-MR mammography-guided breast conserving surgery after neoadjuvant chemotherapy: clinical results and future perspectives with reference to FDG-PET. Breast Cancer 8: 351–354 11791129 10.1007/BF02967536

[bib31] NHSBSP publication No. 3 (1995) Pathology Reporting in Breast Cancer Screening. Sheffield: National Coordinating Group for Breast Screening Pathology

[bib32] Padhani A (2002) Functional MRI for anticancer therapy assessment. Eur H Cancer 38: 2116–2127 10.1016/s0959-8049(02)00388-x 12387837

[bib33] Partridge SC, Gibbs JE, Lu Y, Esserman LJ, Sudilovsky D, Hylton NM (2002) Accuracy of MR imaging for revealing residual breast cancer in patients who have undergone neoadjuvant chemotherapy. AJR Am J Roentgenol 179: 1193–1199 12388497 10.2214/ajr.179.5.1791193

[bib34] Peto R, Boreham J, Clarke M, Davies C, Beral V (2000) UK and USA breast cancer deaths down 25% in year 2000 at ages 20–69 years. Lancet 355: 1822 10.1016/S0140-6736(00)02277-7 10832853

[bib35] Pusztai L, Atyers M, Simmons F, Damokosh A, Hess K, Valero V, Clark E, Ross J, Hortobagyi GN, Stec J (2003) Emerging science: prospective validation of gene expression profiling based on prediction of complete pathologic response to neoadjuvant paclitaxel/FAC chemotherapy in breast cancer. J Clin Oncol 22, Proc. Asco. 10.1200/JCO.2004.05.166 15136595

[bib36] Rieber A, Brambs HJ, Gabelmann A, Heilmann V, Kreienberg R, Kuhn T (2002) Breast MRI for monitoring response of primary breast cancer to neo-adjuvant chemotherapy. Eur Radiol 12: 1711–1719 12111062 10.1007/s00330-001-1233-x

[bib37] Rieber A, Zeitler H, Rosenthal H, Gorich J, Kreienberg R, Brambs HJ, Tomczak R (1997) MRI of breast cancer: influence of chemotherapy on sensitivity. Br J Radiol 70: 452–458 9227225 10.1259/bjr.70.833.9227225

[bib38] Shuman WP, Griffin BR, Haynor DR, Johnson JS, Jones DC, Cromwell LD, Moss AA (1985a) MR imaging in radiation therapy planning. Work in progress. Radiology 156: 143–147 4001401 10.1148/radiology.156.1.4001401

[bib39] Shuman WP, Griffin BR, Yoshy CS, Listerud JA, Mack LA, Rowberg AH, Moss AA (1985b) The impact of CT CORRELATE ScoutView images on radiation therapy planning. AJR Am J Roentgenol 145: 633–638 3875266 10.2214/ajr.145.3.633

[bib40] Sotiriou C, Powles TJ, Dowsett M, Jazaeri AA, Feldman AL, Assersohn L, Gadisetti C, Libutti SK, Liu ET (2002) Gene expression profiles derived from fine needle aspiration correlate with response to systemic chemotherapy in breast cancer. Breast Cancer Res 4: R3 12052255 10.1186/bcr433 PMC111028

[bib41] Therasse P, Arbuck S, Eisenhauer E, Wanders J, Kaplan R, Rubinstein L, Verweij J, Glabbeke M, van Oosterom A, Christina M, Gwyther S (2000) New guidelines to evaluate the response to treatment in solid tumors. European Organization for Research and Treatment of Cancer, National Cancer Institute of the United States, National Cancer Institute of Canada. J Natl Cancer Inst 92: 205–216 10655437 10.1093/jnci/92.3.205

[bib42] Tiling R, Linke R, Untch M, Richter A, Fieber S, Brinkbaumer K, Tatsch K, Hahn K (2001) 18F-FDG PET and 99mTc-sestamibi scintimammography for monitoring breast cancer response to neoadjuvant chemotherapy: a comparative study. Eur J Nucl Med 28: 711–720 11440031 10.1007/s002590100539

[bib43] Trecate G, Ceglia E, Stabile F, Tesoro-Tess JD, Mariani G, Zambetti M, Musumeci R (1998) Locally advanced breast tumors. Role of magnetic resonance in the assessment of response to preoperative therapy and of neoplastic residue before the operation. Radiol Med (Torino) 95: 449–455 9687919

[bib44] Tsuboi N, Ogawa Y, Inomata T, Yoshida D, Yoshida S, Moriki T, Kumon M (1999) Changes in the findings of dynamic MRI by preoperative CAF chemotherapy for patients with breast cancer of stage II and III: pathologic correlation. Oncol Rep 6: 727–732 10373646 10.3892/or.6.4.727

[bib45] Valero V, Buzdar AU, McNeese M, Singletary E, Hortobagyi GN (2002) Primary chemotherapy in the treatment of breast cancer: the University of Texas M D Anderson Cancer Center experience. Clin Breast Cancer 3(Suppl 2): S63–S68 12435294 10.3816/cbc.2002.s.014

[bib46] Vinnicombe SJ, MacVicar AD, Guy RL, Sloane JP, Powles TJ, Knee G, Husband JE (1996) Primary breast cancer: mammographic changes after neoadjuvant chemotherapy, with pathologic correlation. Radiology 198: 333–340 8596827 10.1148/radiology.198.2.8596827

[bib47] Weatherall PT, Evans GF, Metzger GJ, Saborrian MH, Leitch AM (2001) MRI *vs* histologic measurement of breast cancer following chemotherapy: comparison with X-ray mammography and palpation. J Magn Reson Imaging 13: 868–875 11382946 10.1002/jmri.1124

[bib48] Wilson R, Asbury D, Cooke J, Michell M, Patnick J (2001) Clinical Guidelines for Breast Cancer Screening Assessment. Vol. 49. pp. 32 Sheffield: NHSBSP

[bib49] Wolmark N, Wang J, Mamounas E, Bryant J, Fisher B (2001) Preoperative chemotherapy in patients with operable breast cancer: nine-year results from National Surgical Adjuvant Breast and Bowel Project B-18. J Natl Cancer Inst Monogr 30: 96–102 10.1093/oxfordjournals.jncimonographs.a003469 11773300

